# The clinical application of ctDNA to predict response to neoadjuvant chemoradiotherapy in patients with locally-advanced rectal cancer

**DOI:** 10.1186/s40364-023-00521-5

**Published:** 2023-09-19

**Authors:** Parmida Sadat Pezeshki, Reza Ghalehtaki

**Affiliations:** 1 School of Medicine, Tehran University of Medical Sciences, Tehran, Iran; 2 Radiation Oncology Research Center, Cancer Research Institute, Tehran University of Medical Sciences, Qarib St., Keshavarz Blvd, Tehran, Iran; 3 Department of Radiation Oncology, Cancer Institute, IKHC, Tehran University of Medical Sciences, Tehran, Iran

## Abstract

Colorectal cancer is a major cause of cancer-related deaths worldwide. A third of colorectal cancers reside in the rectum. Many patients with rectal cancer present in the locally-advanced stage which needs multi-modality therapy usually starting with neoadjuvant chemo-radiotherapy followed by surgery and adjuvant systemic chemotherapy. Total neoadjuvant therapy, defined as the preoperative administration of both neoadjuvant chemoradiotherapy and systemic chemotherapy is also an evolving treatment that can be delivered if indications for preoperative chemotherapy exist. Identifying biomarkers to predict response to neoadjuvant therapy, can improve patient selection for a non-surgical, active surveillance approach. Circulating tumor DNA (ctDNA) can be detected in about 75% of patients with locally-advanced rectal cancer (LARC) at the baseline and in about 15–20% of patients in the post-neoadjuvant, or postoperative setting. ctDNA clearance rate after delivering neoadjuvant chemoradiotherapy, or integrating baseline ctDNA with other conventional markers of clinical response can be a promising marker to select and monitor patients on the “watch and wait” approach. In this article, we aimed to integrate the recent findings and provide a unique insight into the utilization of preoperative ctDNA to predict clinical response in patients with LARC. We also sought to highlight the potential areas for future research in this field. Further studies with a larger number of participants from diverse populations and settings are needed to increase external validity of such investigations and determine the role of ctDNA in guiding clinical decisions and management of patients with LARC.

To the editor,

Detection of tumor-specific biomarkers with acceptable predictive value has been the core strategy in the pursuit of precision medicine. The biomarker analysis is often conducted on conventional tumor biopsy samples which don’t represent the heterogeneity and dynamic evolution of the tumors. Alternatively, liquid biopsies bring about precision while being a non-invasive modality to comprehend the dynamics of tumor burden. Given the fact that by novel strategies of neoadjuvant chemoradiotherapy (nCRT), a higher number of patients with locally-advanced rectal cancer (LARC) will experience a clinical complete response (cCR) to nCRT, a promising marker to stratify patients based on their response would be of value. Significant studies, such as Galaxy, DYNAMIC, IDEA, CIRCULATE, and COBRA evaluated patients with CRC, specifically colon cancer. Thus, most evidence regarding LARC patients is from smaller studies with limited participants, diminishing their statistical power and limiting conclusions from them.

ctDNA can be detected in more than 75% of patients with LARC before receiving nCRT [1–5]. This detection rate diminishes to 15–20% between the termination of the nCRT and surgery [1, 4, 5]. Despite this considerable detection rate, reports on the predictive value of preoperative ctDNA for long-term oncological outcomes, such as recurrence-free (RFS) or metastasis-free survival (MFS), have been heterogeneous. ctDNA analysis of the preoperative samples seems to be more predictive when taken in the post- than in the pre-nCRT window (Table 1). For instance, Tie et al. failed to report baseline ctDNA as a prognostic marker of RFS [3]. However, post-nCRT preoperative ctDNA was predictive of RFS. In another study, the detection of two or more mutations, in comparison to less than two mutations, at the ctDNA analysis was related to worse disease-free survival (DFS) [6]. Altogether, it seems that even the dynamics of ctDNA can be a more valuable long-term prognostic marker than its presence at baseline. In the study by Khakoo et al., persistent positive ctDNA before, during, and after neoadjuvant nCRT was a more accurate predictor of worse MFS than positive ctDNA at only post-neoadjuvant window.

**Table 1:** Studies investigating preoperative ctDNA in patients with rectal tumors

	Study population	ctDNA detection rate	Primary outcome	HR (CI); P-value	Setting
Tie et al. 2019 [3]	159 pts, T3/4, N + LARC	77%	RFS	1.1 (0.42-3.0); P = 0.823	Pre-neoadjuvant
		8.3%		6.6 (2.6–17); P < 0.001	Post-neoadjuvant, Preoperative
Kotani D et al. 2023 [2]	1039 pts, Stage II-IV resectable CRC	91%	DFS	0.89 (0.55–1.4); P = 0.620	Preoperative
Zhou et al. 2021 [5]	109 pts, LARC	75%	MFS	NA (NA); P = 0.03	Pre-neoadjuvant
		15.6%		6.635 (1.24–35.50); P < 0.001	During-neoadjuvant
		10.5%		19.82 (2.029–193.7); P < 0.001	Post-neoadjuvant, Preoperative
Vidal et al. 2021 [4]	180 pts, LARC	83%	DFS, OS	NA (NA); P = 0.59, NA (NA); P = 0.38	Pre-neoadjuvant
		15%		4; P = 0.033, 23; *P* < 0.0001	Post-neoadjuvant, Preoperative
Murahashi et al. 2020 [8]	85 pts, LARC	57.6%	pCR	Change in ctDNA more than 80%: 7.4 (1.2–144), P = 0.0276	Pre-neoadjuvant
		22.3%			Post-neoadjuvant, Preoperative
Pazdirec et al. 2020 [12]	33 pts, LARC, Stage II/III	21.1%	DFS, OS	NA (NA); P = 0.015, NA (NA); P = 0.01	Pre-neoadjuvant
Roesel et al. 2022 [13]	23 pts, LARC	87%	pCR, Recurrence	NA (NA); P = 0.45, NA (NA); P = 0.54	Pre-neoadjuvant
		17.4%		NA	4 weeks post-neoadjuvant
		13%		NA	7 weeks post-neoadjuvant
		29%		NA (NA); P = 1, NA (NA); P = 0.61	16 weeks post-neoadjuvant (preoperative)
Khakoo et al. 2020 [1]	47 pts, LARC	74.4%	MFS	2.1 (0.5–9.6); P = 0.33	Pre-neoadjuvant
		21.3%		2.6 (0.9–8.1); P = 0.09	On-neoadjuvant
		21.3%		7.1 (2.4–21.5); P < 0.001	Post-neoadjuvant

CRC: colorectal cancer, DFS: disease-free survival, LARC: locally-advanced rectal cancer, OS: overall survival, MFS: metastasis-free survival, NA: not available, pCR: pathological complete response, RFS: regression-free survival

Considering the recent paradigm in multi-modality management of rectal cancer, to improve the value of the current clinically available predictors of the pathological complete response (pCR) prior to surgery, studies have investigated the role of ctDNA as a marker of pCR. ctDNA can also be a promising marker of response and local recurrence in patients who are on an “active surveillance” or “watch and wait” approach and do not undergo surgery after the completion of nCRT [7]. ctDNA change as a function of nCRT can be an independent predictor for pCR [8, 9]. In the aforementioned study by Khakoo et al. [1], post-neoadjuvant ctDNA, in contrast to baseline and during-neoadjuvant ctDNA, was significantly associated with MRI-defined tumor regression grade (MRI-TRG). Wang et al. reported that integrating ctDNA levels, clearance, and the MRI-TRG can provide the optimal predictive value for pCR (Area under the curve (AUC) = 0.886) compared to either ctDNA (AUC = 0.818) or MRI (AUC = 0.729) alone [9]. Another study suggested that a combination of a positive baseline ctDNA and MRI-defined extramural venous invasion can be highly associated with poor response [6]. Among the non-responders in this study, two patients had TP53, one had KRAS, and one had both KRAS and TP53 mutations in the baseline liquid biopsy sample [6]. KRAS and its combination with TP53 mutation have been reported to be associated with radioresistance in patients with CRC [10]. ctDNA clearance of 80% or more was also reported to be an independent predictor of pCR, whereas none of the pre-nCRT and pre-surgical ctDNA levels were associated with the pCR [8]. Among the 39 non-responders evaluated in this study, an increase in the mutation allele frequency (MAF) was observed in only 7 patients. This is in contrast to the responders’ group, where only 1 out of 12 patients exhibited an increase in MAF. It is noteworthy that a significant proportion of non-responders demonstrated a reduction in MAF of ctDNA, albeit this proportion was markedly higher in the responders group. This highlights the limitations of the validity of ctDNA in predicting the response to nCRT. These data suggest that serial ctDNA monitoring, its dynamics, and mutational profile, might be useful additions to the conventional clinical assessment, such as MRI-TRG, to identify optimal candidates for non-operative management.

As mentioned before, currently available data on the value of ctDNA in predicting cCR following nCRT in LARC is mainly based on small-scale studies. Larger LARC-focused studies could clarify ctDNA’s role in clinical decision-making. Enhanced predictive accuracy could be achieved through tissue-directed liquid biopsies, the use of advanced NGS panels for ctDNA detection, and the integration of other tumor biomarkers. Utilizing advanced analytical techniques like machine or deep learning could further improve the predictive performance of these complex models. Given the constraints of current imaging and staging methods, it is possible to categorize subgroups of patients with LARC by utilizing ctDNA-based assessments. This allows us to recommend risk-adjusted therapies and investigate the corresponding treatment outcomes. External validation is also a critical factor in assessing the robustness and applicability of the discussed findings in this article. Further multi-center trials across diverse populations and settings would render these results more generalizable and reliable. They can also lead to the identification of potential sources of bias or heterogeneity that may affect the predictive performance of the models based on ctDNA detection.

ctDNA has been also studied as a beneficial tool to select and monitor patients in a timely manner for targeted therapies, i.e. anti-EGFR or anti-PD-1/PD-L1. Serial ctDNA samples would be a convenient tool to comprehend cancer cells’ evolution towards secondary resistance while on targeted therapies. However, their implementation in this setting has been mostly investigated in metastatic CRC and not localized tumors. This could be explained by the fact that the emerging benefit of targeted therapies in the neoadjuvant setting for localized tumors such as LARC is only recently being investigated [11].

## Data Availability

Data sharing is not applicable to this article as no datasets were generated or analysed during the current study.
